# Crystal structure of bis­(acetato-κ*O*)di­aqua­(2,2′-bi­pyridine-κ^2^ *N*,*N*′)manganese(II)

**DOI:** 10.1107/S1600536814017814

**Published:** 2014-08-13

**Authors:** Natarajan Saravanan, Parasuraman Selvam

**Affiliations:** a National Centre for Catalysis Research, Department of Chemistry, Indian Institute of Technology-Madras, Chennai 600 036, India

**Keywords:** crystal structure, acetate, 2,2′-bi­pyridine, monomeric manganese(II) complex

## Abstract

In the title monomeric manganese(II) complex, [Mn(CH_3_COO)_2_(C_10_H_8_N_2_)(H_2_O)_2_], the metal ion is coordinated by a bidentate 2,2′-bi­pyridine (bpy) ligand, two water mol­ecules and two axial acetate anions, resulting in a highly distorted octa­hedral environment. The aqua ligands are stabilized by the formation of strong intra­molecular hydrogen bonds with the uncoordinated acetate O atoms, giving rise to pseudo-bridging arrangement of the terminal acetate groups. In the crystal, the mol­ecules form [010] zigzag chains *via* O—H⋯O hydrogen bonds involving the aqua ligands and acetate O atoms. Further, the water and bpy ligands are *trans* to each other, and are arranged in an off-set fashion showing inter­molecular π–π stacking between nearly parallel bi­py rings, the centroid–centroid separations being 3.8147 (12) and 3.9305 (13) Å.

## Related literature   

For complexes with the same ligands as the title complex, see: Chen *et al.* (1995 ▶); Carballo *et al.* (2001 ▶); Hu *et al.* (2011 ▶); Ye *et al.* (1998 ▶); Zhao *et al.* (2009 ▶). For ionic radii, see: Shannon (1976 ▶).
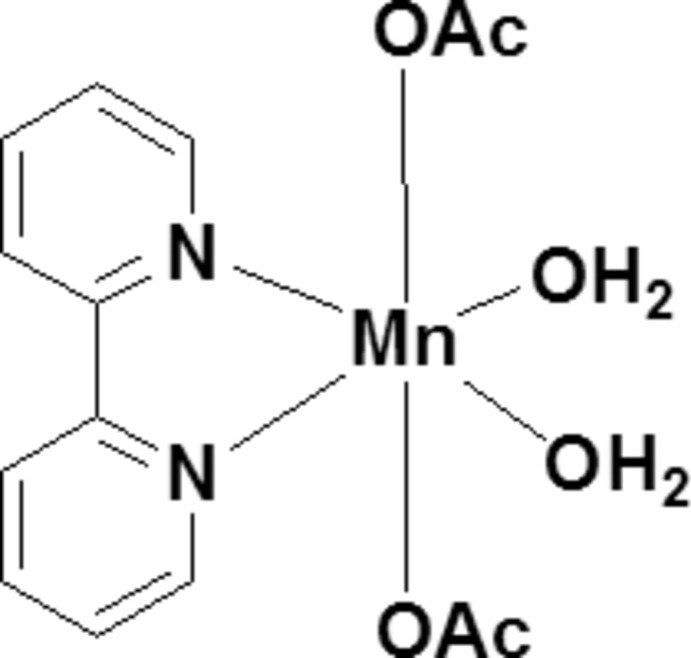


## Experimental   

### Crystal data  

[Mn(C_2_H_3_O_2_)_2_(C_10_H_8_N_2_)(H_2_O)_2_]
*M* *_r_* = 365.24
Monoclinic, 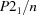

*a* = 12.8494 (8) Å
*b* = 8.1434 (5) Å
*c* = 15.5918 (10) Å
β = 98.926 (2)°
*V* = 1611.73 (18) Å^3^
*Z* = 4
Mo *K*α radiation
μ = 0.85 mm^−1^
*T* = 296 K
0.30 × 0.25 × 0.16 mm

### Data collection  

Bruker APEXII CCD diffractometer
Absorption correction: multi-scan (*SADABS*; Bruker, 2004 ▶) *T*_min_ = 0.775, *T*_max_ = 0.873
11092 measured reflections
2820 independent reflections
2469 reflections with *I* > 2σ(*I*)
*R*_int_ = 0.023

### Refinement  

*R*[*F* ^2^ > 2σ(*F* ^2^)] = 0.029
*wR*(*F* ^2^) = 0.076
*S* = 1.06
2820 reflections
226 parameters
H atoms treated by a mixture of independent and constrained refinement
Δρ_max_ = 0.26 e Å^−3^
Δρ_min_ = −0.21 e Å^−3^

### 

Data collection: *APEX2* (Bruker, 2004 ▶); cell refinement: *SAINT-Plus* (Bruker, 2004 ▶); data reduction: *SAINT-Plus*; program(s) used to solve structure: *SHELXS97* (Sheldrick 2008 ▶); program(s) used to refine structure: *SHELXL2013* (Sheldrick, 2008 ▶); molecular graphics: *SHELXTL* (Sheldrick 2008 ▶); software used to prepare material for publication: *SHELXTL*.

## Supplementary Material

Crystal structure: contains datablock(s) global, I. DOI: 10.1107/S1600536814017814/gw2147sup1.cif

Structure factors: contains datablock(s) I. DOI: 10.1107/S1600536814017814/gw2147Isup2.hkl

Supporting information file. DOI: 10.1107/S1600536814017814/gw2147Isup3.cdx

**Fig. 1.**
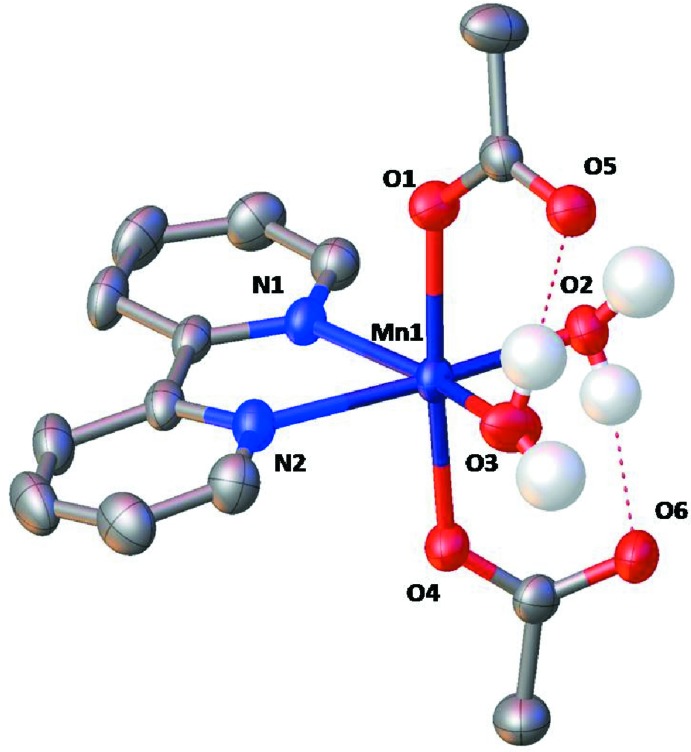
ORTEP of the molecule with atoms represented as 30% probability ellipsoids.

**Fig. 2.**
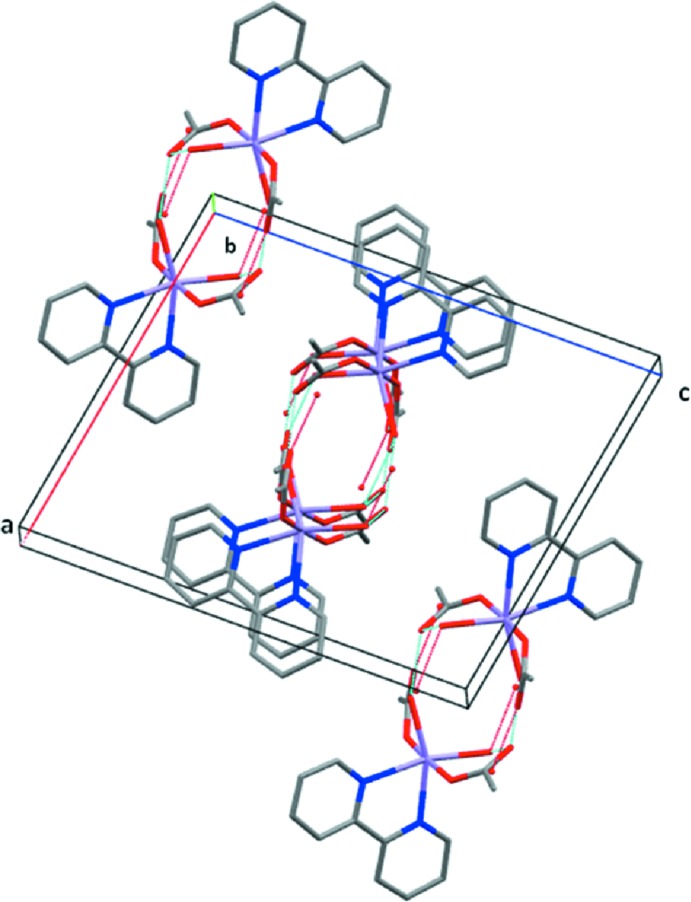
Molecular packing of complex 1 viewed along b axis.

ORTEP . DOI: 10.1107/S1600536814017814/gw2147fig1.tif
*ORTEP* of the mol­ecule with atoms represented as 30% probability ellipsoids.

b . DOI: 10.1107/S1600536814017814/gw2147fig2.tif
Mol­ecular packing of complex 1 viewed along *b* axis.

CCDC reference: 1006361

Additional supporting information:  crystallographic information; 3D view; checkCIF report

## Figures and Tables

**Table 1:** Selected bond lengths (Å)

Mn1—O4	2.1634 (15)
Mn1—O1	2.1736 (15)
Mn1—O3	2.1918 (15)
Mn1—O2	2.2038 (16)
Mn1—N1	2.2679 (15)
Mn1—N2	2.2869 (16)

**Table 2:** Hydrogen-bond geometry (Å, °)

*D*—H⋯*A*	*D*—H	H⋯*A*	*D*⋯*A*	*D*—H⋯*A*
O2—H1*X*⋯O6^i^	0.80 (3)	2.05 (3)	2.815 (2)	161 (3)
O2—H2*X*⋯O5	0.89 (3)	1.76 (3)	2.636 (2)	169 (2)
O3—H3*X*⋯O6	0.80 (3)	1.90 (3)	2.684 (2)	168 (3)
O3—H4*X*⋯O5^ii^	0.91 (3)	1.84 (3)	2.734 (2)	169 (3)

Symmetry codes: (i) 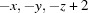; (ii) 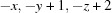.

## supplementary crystallographic information

### S1. Comment

The co-existence of solvent and bpy molecules in certain metal complexes have been reported in several lanthanide (III) complexes containing bpy and carboxylate ligands, (Chen *et al.*, 1995) while the transition metal ions complexes, *e.g.*, Co^II^(bpy)(OAc)_2_(H_2_O)_2_, Ni^II^(bpy)(OAc)_2_(H_2_O)_2_ and Ni^II^(bpy)(pda)_2_(H_2_O)_2_ complexes with mixed bpy, acetate and solvate molecules, is also established (Ye *et al.*, 1998; Carballo *et al.*, 2001; Hu *et al.*, 2011). We describe herein the crystal structure of divalent manganese complex, *viz.* Mn^II^(bpy)(OAc)_2_(H_2_O)_2_ with 2,2'-bipyridine, acetate and solvate molecule as the coordinating ligands, and the intermolecular hydrogen bonding interaction between the coordinated acetates and the solvent molecules.

The crystal structure of the complex 1 is illustrated in Fig. 1. The metal ion in the title complex is coordinated by bidentate bpy ligand, two water molecules and two axial acetate anions coordinated trans to each other resulting in a highly distorted octahedral [MnN2O4] coordination geometry. The Mn—N bond lengths range from 2.2679 (15)–2.2869 (16) Å and the shortest Mn—OAc axial bonds range from 2.163 (15)–2.173 (15) Å, may partially be ascribed to the mutual strong coordination between the anionic ligands to the divalent manganese center, as evidenced by the fact that the pyridine ring coordinates to the metal atom in a non planar fashion with the torsion angle Mn(1)—N(1)—C(2)—C(3) = 170.8°. The Mn—Npy (2.2679 (15) Å); Mn—OAc (2.2038 (16) Å), and Mn—O(w) (2.1918 (15) Å) bonds in 1 are longer than that of the respective Co- and Ni-analogues, viz., Co^II^(bpy)(OAc)_2_(H_2_O)_2_ and Ni^II^(bpy)(OAc)_2_(H_2_O)_2_, with symmetrical M—N bonds (Co—Npy= 2.122 (14) Å); Ni—Npy= 2.069 (2) Å). Similarly, the Mn—O(w) and Mn—OAc bonds in Mn^II^(bpy)(OAc)_2_(H_2_O)_2_ are also longer than the corresponding Co- and Ni-analogues (Co—OAc = 2.097 (13) Å; Ni—OAc = 2.079 (2) Å); Co—O(w) = 2.125 (13) Å; Ni—O(w) = 2.082 (2) Å), revealing the largest repulsion of hexacoordinated divalent manganese center due to its larger radius (0.97 Å), (Shannon, 1976) as compared to the analogous M(bpy)(OAc)_2_(H_2_O)_2_ complexes containing divalent cobalt (0.89 Å) and divalent nickel (0.83 Å). The most distorted O(2)—Mn—O(3) = 102.72° bond angles from an idealized octahedron resulting from the water molecules coordinated trans to bipyridine moiety.

In crystal lattice of Mn^II^(bpy)(OAc)_2_(H_2_O)_2_, the coordinated water molecule showing significant intermolecular hydrogen bonding interaction with axially coordinated acetate anion and bpy ligands are arranged in a stacking interaction with close interplanar contacts of ca. 3.40 Å and the separation between the planes of each pair of adjacent pyridyl rings is ca. 8.14 Å (Fig. 2). Such relatively short interplanar contacts are indicative of extensive π–π stacking interaction between the pyridine rings. The existence of intermolecular hydrogen bonding and stacking interaction of bpy ligands is very important in stabilizing molecular structure in the solid state as shown in Fig. 1 and Fig. 2. A similar behavior was also noticed for mononuclear nickel complexes assembled into two-dimensional networks via hydrogen bonds and showing significant π–π stacking interactions where the close interchain bipyridyl groups, being a rranged in an off-set fashion, have an average face-to-face distance of 3.44 Å for Ni(bipy)(OAc)_2_(H_2_O)_2_ and 3.60 Å for Ni(dmbipy)(OAc)_2_(H_2_O)_2_ (Ye *et al.* 1998).

### S2. Experimental

Synthesis of MnII(bpy)(OAc)_2_(H_2_O)_2_: Manganous acetate tetrahydrate (0.245 g, 1.0 mmol) was dissolved in an dry methanol (20 ml) and then an methanolic solution (10 ml) of 2,2'-bipyridine (0.156 g, 1.0 mmol) was added drop-wise with continuous stirring. The resulting mixture was refluxed for an hour and then filtered to remove the brownish precipitate. The light yellow filtrate was allowed to stand undisturbed for two weeks or so at room temperature, during which brown crystals of 1, suitable for X-ray diffraction analysis, were deposited in *ca* 60% yield (based on Mn). Anal. Calcd. (%) for C_14_H_20_MnN_2_O_6_: C, 45.79; H, 5.49; N, 7.63. Found (%): C, 45.34; H, 5.37; N, 7.66.

### S3. Refinement

All H atoms were added according to theoretical models, assigned isotropic displacement parameters and allowed to ride on their respective parent atoms[C—H=0.93–0.97%A and *U*_iso_=1.2Ueq].

### Crystal data

**Table:**

[Mn(C_2_H_3_O_2_)_2_(C_10_H_8_N_2_)(H_2_O)_2_]	*F*(000) = 756
*M**_r_* = 365.24	*D*_x_ = 1.505 Mg m^−^^3^
Monoclinic, *P*2_1_/*n*	Mo *K*α radiation, λ = 0.71073 Å
*a* = 12.8494 (8) Å	Cell parameters from 6258 reflections
*b* = 8.1434 (5) Å	θ = 2.2–28.2°
*c* = 15.5918 (10) Å	µ = 0.85 mm^−^^1^
β = 98.926 (2)°	*T* = 296 K
*V* = 1611.73 (18) Å^3^	Rectangular, brown
*Z* = 4	0.30 × 0.25 × 0.16 mm

### Data collection

**Table:**

Bruker APEXII CCD diffractometer	2469 reflections with *I* > 2σ(*I*)
φ and ω scans	*R*_int_ = 0.023
Absorption correction: multi-scan (*SADABS*; Bruker, 2004)	θ_max_ = 25.0°, θ_min_ = 1.9°
*T*_min_ = 0.775, *T*_max_ = 0.873	*h* = −15→15
11092 measured reflections	*k* = −9→8
2820 independent reflections	*l* = −18→18

### Refinement

**Table:**

Refinement on *F*^2^	0 restraints
Least-squares matrix: full	H atoms treated by a mixture of independent and constrained refinement
*R*[*F*^2^ > 2σ(*F*^2^)] = 0.029	*w* = 1/[σ^2^(*F*_o_^2^) + (0.0349*P*)^2^ + 0.7672*P*] where *P* = (*F*_o_^2^ + 2*F*_c_^2^)/3
*wR*(*F*^2^) = 0.076	(Δ/σ)_max_ = 0.001
*S* = 1.06	Δρ_max_ = 0.26 e Å^−^^3^
2820 reflections	Δρ_min_ = −0.21 e Å^−^^3^
226 parameters	

### Special details

**Table:**

Geometry. All e.s.d.'s (except the e.s.d. in the dihedral angle between two l.s. planes) are estimated using the full covariance matrix. The cell e.s.d.'s are taken into account individually in the estimation of e.s.d.'s in distances, angles and torsion angles; correlations between e.s.d.'s in cell parameters are only used when they are defined by crystal symmetry. An approximate (isotropic) treatment of cell e.s.d.'s is used for estimating e.s.d.'s involving l.s. planes.

### Fractional atomic coordinates and isotropic or equivalent isotropic displacement parameters (Å^2^)

**Table:**

	*x*	*y*	*z*	*U*_iso_*/*U*_eq_	
C1	0.40953 (16)	0.3913 (3)	1.13971 (13)	0.0389 (5)	
H1	0.3553	0.4152	1.1709	0.047*	
C2	0.51018 (17)	0.4448 (3)	1.17221 (15)	0.0476 (6)	
H2	0.5238	0.5018	1.2244	0.057*	
C3	0.58959 (17)	0.4107 (3)	1.12467 (15)	0.0501 (6)	
H3	0.6579	0.4469	1.1439	0.060*	
C4	0.56718 (15)	0.3231 (3)	1.04872 (14)	0.0428 (5)	
H4	0.6203	0.2987	1.0165	0.051*	
C5	0.46481 (14)	0.2714 (2)	1.02057 (13)	0.0316 (4)	
C6	0.43430 (14)	0.1760 (3)	0.93939 (12)	0.0327 (4)	
C7	0.50731 (16)	0.1105 (3)	0.89147 (14)	0.0437 (6)	
H7	0.5791	0.1244	0.9102	0.052*	
C8	0.47254 (19)	0.0249 (3)	0.81621 (15)	0.0506 (6)	
H8	0.5206	−0.0184	0.7835	0.061*	
C9	0.36604 (19)	0.0042 (3)	0.78996 (14)	0.0500 (6)	
H9	0.3408	−0.0529	0.7393	0.060*	
C10	0.29791 (17)	0.0702 (3)	0.84067 (13)	0.0434 (5)	
H10	0.2260	0.0555	0.8233	0.052*	
C11	0.11250 (15)	0.5562 (3)	0.92173 (12)	0.0327 (5)	
C12	0.11895 (19)	0.7383 (3)	0.90939 (18)	0.0539 (6)	
H12A	0.0999	0.7643	0.8489	0.081*	
H12B	0.1896	0.7748	0.9293	0.081*	
H12C	0.0715	0.7924	0.9421	0.081*	
C13	0.18311 (15)	−0.0662 (3)	1.12096 (13)	0.0358 (5)	
C14	0.21073 (19)	−0.2375 (3)	1.15213 (17)	0.0507 (6)	
H14A	0.2282	−0.3022	1.1049	0.076*	
H14B	0.1516	−0.2858	1.1736	0.076*	
H14C	0.2700	−0.2340	1.1979	0.076*	
Mn1	0.21987 (2)	0.23631 (4)	1.00781 (2)	0.03042 (11)	
N1	0.38666 (12)	0.3072 (2)	1.06586 (10)	0.0314 (4)	
N2	0.32980 (12)	0.1546 (2)	0.91379 (10)	0.0341 (4)	
O1	0.19541 (10)	0.48188 (18)	0.95383 (9)	0.0409 (4)	
O2	0.08066 (12)	0.1747 (2)	0.91193 (12)	0.0501 (4)	
O3	0.14983 (11)	0.3191 (2)	1.11986 (10)	0.0396 (4)	
O4	0.23998 (10)	0.00016 (18)	1.07180 (9)	0.0399 (3)	
O5	0.02567 (10)	0.48655 (18)	0.89735 (10)	0.0411 (4)	
O6	0.10520 (12)	0.0026 (2)	1.14539 (12)	0.0529 (4)	
H2X	0.056 (2)	0.276 (4)	0.9014 (17)	0.063 (9)*	
H1X	0.034 (2)	0.109 (4)	0.9040 (18)	0.070 (10)*	
H4X	0.096 (2)	0.392 (4)	1.1099 (17)	0.074 (9)*	
H3X	0.131 (2)	0.230 (4)	1.1320 (17)	0.054 (9)*	

### Atomic displacement parameters (Å^2^)

**Table:**

	*U*^11^	*U*^22^	*U*^33^	*U*^12^	*U*^13^	*U*^23^
C1	0.0341 (11)	0.0412 (13)	0.0416 (11)	−0.0004 (9)	0.0067 (9)	0.0019 (10)
C2	0.0427 (13)	0.0515 (15)	0.0458 (12)	−0.0095 (11)	−0.0023 (10)	−0.0010 (11)
C3	0.0290 (11)	0.0636 (17)	0.0547 (14)	−0.0136 (11)	−0.0033 (10)	0.0107 (12)
C4	0.0204 (9)	0.0604 (15)	0.0478 (13)	−0.0004 (10)	0.0065 (9)	0.0129 (11)
C5	0.0212 (9)	0.0355 (12)	0.0385 (11)	0.0021 (8)	0.0056 (8)	0.0123 (9)
C6	0.0247 (9)	0.0384 (12)	0.0359 (10)	0.0054 (9)	0.0074 (8)	0.0114 (9)
C7	0.0297 (11)	0.0552 (15)	0.0485 (12)	0.0116 (10)	0.0135 (9)	0.0120 (11)
C8	0.0550 (14)	0.0572 (16)	0.0443 (13)	0.0194 (12)	0.0231 (11)	0.0063 (11)
C9	0.0593 (15)	0.0561 (16)	0.0358 (11)	0.0080 (12)	0.0115 (10)	0.0001 (11)
C10	0.0378 (11)	0.0534 (15)	0.0383 (11)	−0.0008 (11)	0.0039 (9)	0.0017 (10)
C11	0.0308 (11)	0.0349 (12)	0.0335 (10)	0.0036 (9)	0.0088 (8)	0.0018 (9)
C12	0.0463 (14)	0.0355 (14)	0.0749 (17)	0.0024 (10)	−0.0067 (12)	0.0028 (12)
C13	0.0258 (10)	0.0354 (12)	0.0454 (12)	−0.0028 (9)	0.0029 (9)	0.0009 (9)
C14	0.0516 (14)	0.0412 (14)	0.0615 (15)	0.0047 (11)	0.0155 (12)	0.0103 (11)
Mn1	0.01912 (16)	0.03294 (19)	0.03953 (19)	0.00163 (12)	0.00552 (12)	0.00237 (13)
N1	0.0227 (8)	0.0343 (10)	0.0371 (9)	0.0002 (7)	0.0048 (7)	0.0050 (7)
N2	0.0268 (8)	0.0405 (11)	0.0355 (9)	0.0034 (7)	0.0065 (7)	0.0054 (8)
O1	0.0266 (7)	0.0363 (9)	0.0582 (9)	0.0029 (6)	0.0015 (6)	0.0088 (7)
O2	0.0291 (8)	0.0373 (10)	0.0783 (12)	−0.0018 (8)	−0.0089 (8)	−0.0034 (9)
O3	0.0303 (8)	0.0364 (10)	0.0546 (9)	0.0052 (8)	0.0140 (7)	0.0024 (8)
O4	0.0316 (7)	0.0356 (8)	0.0548 (9)	0.0042 (6)	0.0143 (6)	0.0086 (7)
O5	0.0268 (7)	0.0379 (9)	0.0571 (9)	0.0032 (6)	0.0024 (6)	0.0031 (7)
O6	0.0374 (8)	0.0409 (10)	0.0865 (12)	0.0016 (7)	0.0284 (8)	0.0067 (8)

### Geometric parameters (Å, º)

**Table:**

C1—N1	1.333 (3)	C11—O1	1.260 (2)
C1—C2	1.384 (3)	C11—C12	1.499 (3)
C1—H1	0.9300	C12—H12A	0.9600
C2—C3	1.379 (3)	C12—H12B	0.9600
C2—H2	0.9300	C12—H12C	0.9600
C3—C4	1.374 (3)	C13—O6	1.257 (2)
C3—H3	0.9300	C13—O4	1.260 (2)
C4—C5	1.386 (3)	C13—C14	1.501 (3)
C4—H4	0.9300	C14—H14A	0.9600
C5—N1	1.346 (2)	C14—H14B	0.9600
C5—C6	1.485 (3)	C14—H14C	0.9600
C6—N2	1.351 (2)	Mn1—O4	2.1634 (15)
C6—C7	1.393 (3)	Mn1—O1	2.1736 (15)
C7—C8	1.378 (3)	Mn1—O3	2.1918 (15)
C7—H7	0.9300	Mn1—O2	2.2038 (16)
C8—C9	1.376 (3)	Mn1—N1	2.2679 (15)
C8—H8	0.9300	Mn1—N2	2.2869 (16)
C9—C10	1.377 (3)	O2—H2X	0.89 (3)
C9—H9	0.9300	O2—H1X	0.79 (3)
C10—N2	1.340 (3)	O3—H4X	0.91 (3)
C10—H10	0.9300	O3—H3X	0.80 (3)
C11—O5	1.257 (2)		
			
N1—C1—C2	123.1 (2)	O6—C13—O4	123.9 (2)
N1—C1—H1	118.4	O6—C13—C14	118.38 (19)
C2—C1—H1	118.4	O4—C13—C14	117.72 (19)
C3—C2—C1	117.9 (2)	C13—C14—H14A	109.5
C3—C2—H2	121.1	C13—C14—H14B	109.5
C1—C2—H2	121.1	H14A—C14—H14B	109.5
C4—C3—C2	119.60 (19)	C13—C14—H14C	109.5
C4—C3—H3	120.2	H14A—C14—H14C	109.5
C2—C3—H3	120.2	H14B—C14—H14C	109.5
C3—C4—C5	119.5 (2)	O4—Mn1—O1	174.99 (5)
C3—C4—H4	120.3	O4—Mn1—O3	86.57 (6)
C5—C4—H4	120.3	O1—Mn1—O3	88.46 (6)
N1—C5—C4	121.1 (2)	O4—Mn1—O2	97.86 (7)
N1—C5—C6	116.15 (16)	O1—Mn1—O2	83.88 (6)
C4—C5—C6	122.74 (18)	O3—Mn1—O2	102.72 (6)
N2—C6—C7	120.87 (19)	O4—Mn1—N1	90.26 (6)
N2—C6—C5	115.98 (16)	O1—Mn1—N1	89.49 (6)
C7—C6—C5	123.15 (18)	O3—Mn1—N1	94.76 (6)
C8—C7—C6	119.6 (2)	O2—Mn1—N1	161.08 (6)
C8—C7—H7	120.2	O4—Mn1—N2	89.74 (6)
C6—C7—H7	120.2	O1—Mn1—N2	94.94 (6)
C9—C8—C7	119.4 (2)	O3—Mn1—N2	166.23 (6)
C9—C8—H8	120.3	O2—Mn1—N2	90.92 (6)
C7—C8—H8	120.3	N1—Mn1—N2	71.97 (6)
C8—C9—C10	118.2 (2)	C1—N1—C5	118.82 (17)
C8—C9—H9	120.9	C1—N1—Mn1	122.94 (13)
C10—C9—H9	120.9	C5—N1—Mn1	118.11 (13)
N2—C10—C9	123.5 (2)	C10—N2—C6	118.44 (17)
N2—C10—H10	118.3	C10—N2—Mn1	123.85 (13)
C9—C10—H10	118.3	C6—N2—Mn1	117.22 (13)
O5—C11—O1	124.03 (19)	C11—O1—Mn1	131.20 (13)
O5—C11—C12	118.15 (18)	Mn1—O2—H2X	98.4 (17)
O1—C11—C12	117.79 (19)	Mn1—O2—H1X	140 (2)
C11—C12—H12A	109.5	H2X—O2—H1X	111 (3)
C11—C12—H12B	109.5	Mn1—O3—H4X	117.6 (17)
H12A—C12—H12B	109.5	Mn1—O3—H3X	94.8 (19)
C11—C12—H12C	109.5	H4X—O3—H3X	113 (3)
H12A—C12—H12C	109.5	C13—O4—Mn1	128.52 (13)
H12B—C12—H12C	109.5		
			
N1—C1—C2—C3	1.0 (4)	C2—C1—N1—Mn1	−175.33 (17)
C1—C2—C3—C4	−1.4 (4)	C4—C5—N1—C1	−1.2 (3)
C2—C3—C4—C5	0.6 (4)	C6—C5—N1—C1	179.38 (18)
C3—C4—C5—N1	0.8 (3)	C4—C5—N1—Mn1	174.66 (15)
C3—C4—C5—C6	−179.9 (2)	C6—C5—N1—Mn1	−4.7 (2)
N1—C5—C6—N2	8.5 (3)	C9—C10—N2—C6	−0.1 (3)
C4—C5—C6—N2	−170.84 (19)	C9—C10—N2—Mn1	−171.77 (17)
N1—C5—C6—C7	−171.14 (19)	C7—C6—N2—C10	−0.7 (3)
C4—C5—C6—C7	9.5 (3)	C5—C6—N2—C10	179.61 (19)
N2—C6—C7—C8	1.1 (3)	C7—C6—N2—Mn1	171.52 (16)
C5—C6—C7—C8	−179.3 (2)	C5—C6—N2—Mn1	−8.2 (2)
C6—C7—C8—C9	−0.6 (4)	O5—C11—O1—Mn1	−16.3 (3)
C7—C8—C9—C10	−0.2 (4)	C12—C11—O1—Mn1	165.72 (16)
C8—C9—C10—N2	0.5 (4)	O6—C13—O4—Mn1	−4.4 (3)
C2—C1—N1—C5	0.3 (3)	C14—C13—O4—Mn1	175.79 (15)

### Hydrogen-bond geometry (Å, º)

**Table:**

*D*—H···*A*	*D*—H	H···*A*	*D*···*A*	*D*—H···*A*
O2—H1*X*···O6^i^	0.80 (3)	2.05 (3)	2.815 (2)	161 (3)
O2—H2*X*···O5	0.89 (3)	1.76 (3)	2.636 (2)	169 (2)
O3—H3*X*···O6	0.80 (3)	1.90 (3)	2.684 (2)	168 (3)
O3—H4*X*···O5^ii^	0.91 (3)	1.84 (3)	2.734 (2)	169 (3)

Symmetry codes: (i) −*x*, −*y*, −*z*+2; (ii) −*x*, −*y*+1, −*z*+2.
