# Supplementary figures and images for: Crystal structure of bis­(acetato-κO)di­aqua­(2,2′-bi­pyridine-κ2 N,N′)manganese(II)
Source: Acta Crystallogr Sect E Struct Rep Online. 2014 Aug 13;70(Pt 9):m326–7. doi: 10.1107/S1600536814017814 (PMC4186102; doi:10.1107/S1600536814017814)

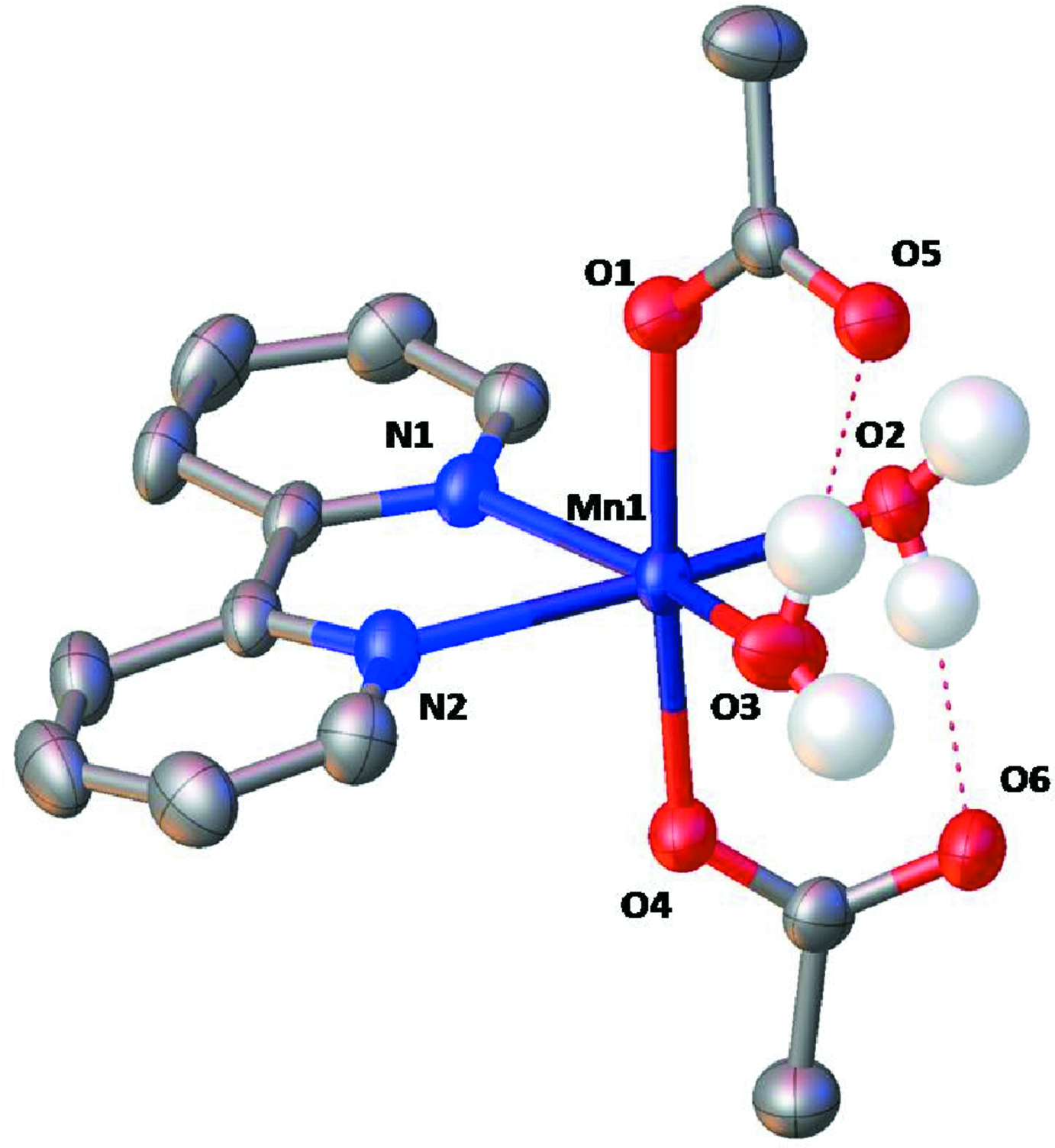

Supplement: Supplementary file 4 [file e-70-0m326-fig1.tif]

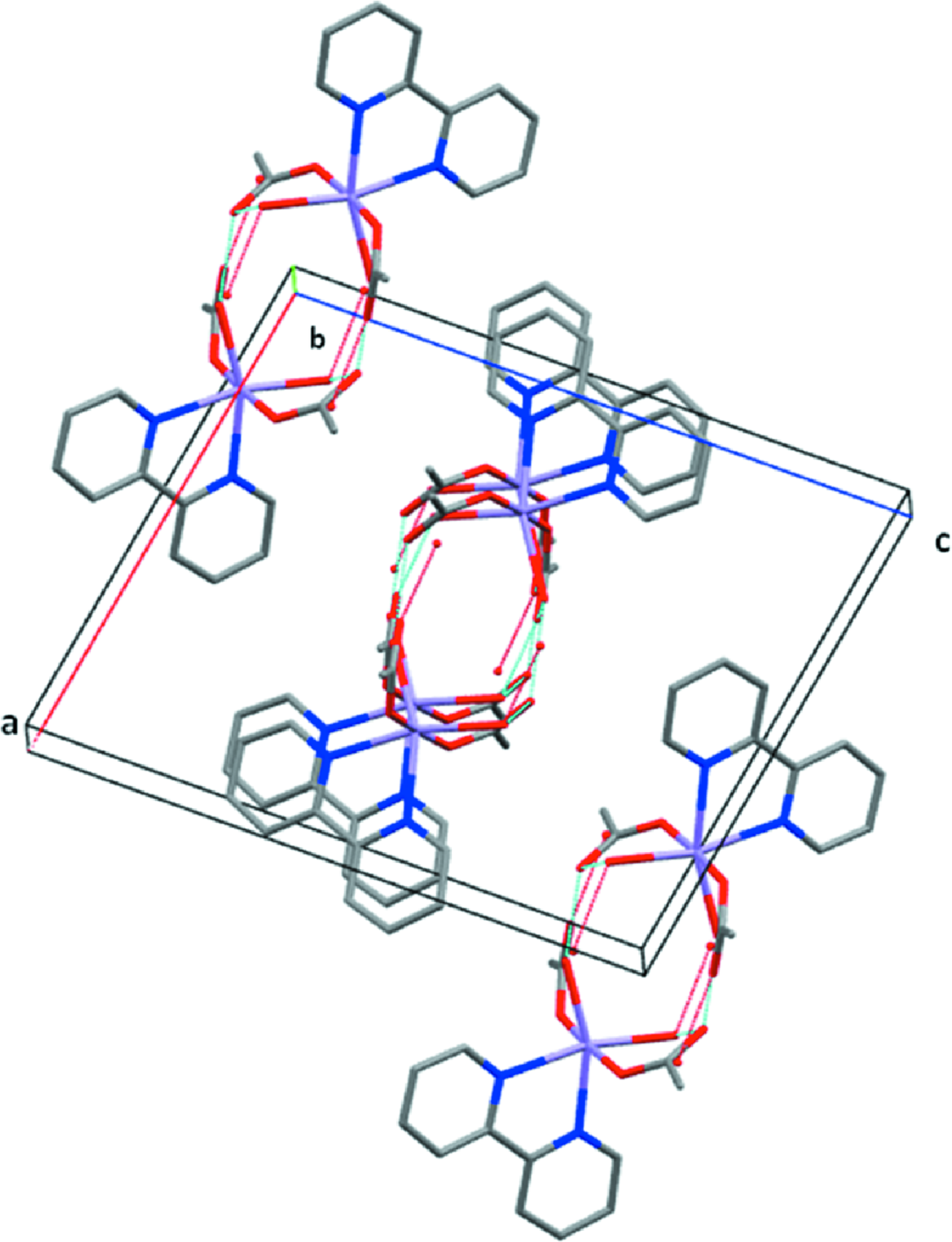

Supplement: Supplementary file 5 [file e-70-0m326-fig2.tif]
